# Flotation Assay With Fluorescence Readout to Study Membrane Association of the Enteroviral Peripheral Membrane Protein 2C

**DOI:** 10.21769/BioProtoc.5261

**Published:** 2025-04-05

**Authors:** Kasturika Shankar, Yuyang Lin, Lars-Anders Carlson

**Affiliations:** 1 Department of Medical Biochemistry and Biophysics, Umeå University, Umeå, Sweden; 2 Wallenberg Centre for Molecular Medicine, Umeå University, Umeå, Sweden; 3 Laboratory for Molecular Infection Medicine Sweden (MIMS), Umeå University, Umeå, Sweden; 4 Umeå Centre for Microbial Research (UCMR), Umeå University, Umeå, Sweden

**Keywords:** Enterovirus, 2C, Lipid, Membrane, Peripheral membrane protein, Flotation assay, Ultracentrifugation

## Abstract

Enteroviruses are abundant pathogens of humans and animals. Their replication is strictly dependent on the conserved, viral AAA+ ATPase 2C. 2C is an oligomerizing, peripheral membrane protein, and its low solubility as recombinant protein has hampered functional studies of the full-length, recombinant protein bound to a membrane. Here, we describe a modification of the classical, ultracentrifugation-based liposome flotation assay optimized to study the interaction of recombinant 2C with membranes and the functions of membrane-bound, full-length recombinant 2C. The assay takes advantage of the high solubility of recombinant 2C while fused to a maltose-binding protein. Removing this solubility-enhancing tag by specific protease cleavage in the presence of liposomes allows 2C to associate with membranes prior to aggregating. Fluorophore labeling of protein and liposomes allows rapid and precise quantitation of 2C’s association with membranes. This assay is adaptable to any peripheral membrane protein that can be fluorophore-labeled and expressed as a solubility-enhancing fusion protein.

Key features

• This protocol extends widely used liposome flotation assays to low-solubility peripheral membrane proteins, such as the enteroviral protein 2C.

• 2C is expressed and purified as a fusion protein with a solubility-enhancing tag, which is cleaved off in the presence of liposomes.

• Fluorophore-labeling of liposomes and protein facilitates quantitative readout of protein association with membranes.

• The protein-conjugated liposomes can also be used for other studies using, e.g., dynamic light scattering, cryo-EM, and enzymatic activity assays.

• The protein-conjugated liposomes can also be used for other studies using, e.g., dynamic light scattering, cryo-EM, and enzymatic activity assays.

## Graphical overview


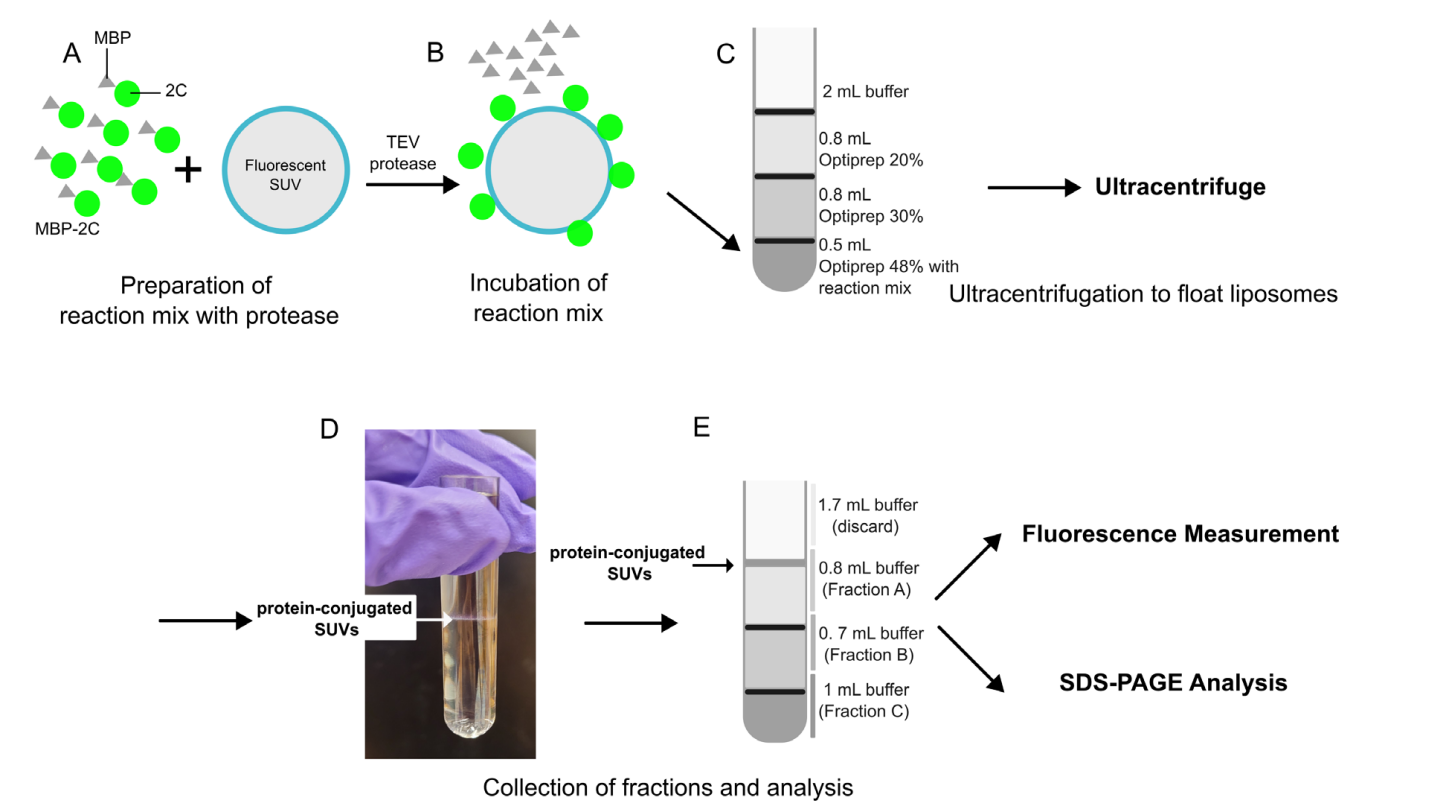


**Overview of flotation assay**

## Background

Peripheral membrane proteins can associate with membranes through interactions with integral membrane proteins, binding to lipid headgroups, or shallow hydrophobic insertion. Understanding the factors that control their membrane association (and dissociation) is often an important step toward elucidating peripheral membrane proteins’ function. However, they frequently have limited solubility, which hampers their study in biochemical assays relying on purified proteins. To circumvent protein insolubility problems, researchers sometimes resort to strategies that increase protein solubility, such as truncation of the membrane-binding part of the protein, expression of the protein fused with a solubility-enhancing tag, or purification in the presence of detergents [1,2]. Though such strategies allow researchers to study the protein, they all involve various compromises. A key protein in the replication of enteroviruses is the peripheral membrane protein 2C, a conserved AAA+ ATPase. 2C has previously been studied using the solubility-enhancing methods described above. Here, we describe a variation of the liposome flotation assay [3] that allows the study of recombinant, full-length poliovirus 2C bound to membranes and the use of these protein-conjugated vesicles for activity assays, cryo-EM, etc. This protocol uses full-length 2C protein, which is purified as a fusion protein with an N-terminal maltose-binding protein (MBP) tag. This tag is proteolytically removed from 2C in the presence of liposomes, which allows the full-length protein to associate with a membrane before precipitating. An additional advantage to this version of the flotation assay includes quantitation through fluorescent labeling of protein and lipid. The protein-coated liposomes can also be used for other assays without involving the flotation step. Limitations of the method include the requirement of a costly ultracentrifuge with a suitable swing-out rotor as well as a fluorimeter or fluorescence-capable plate reader for fluorescence quantitation.

## Materials and reagents

**Reagents**

1. 1-palmitoyl-2-oleoyl-sn-glycero-3-phosphoethanolamine (POPE) (Avanti Polar Lipids, catalog number: 850757P-25 mg)

2. 1-palmitoyl-2-oleoyl-glycero-3-phosphocholine (POPC) (Avanti Polar Lipids, catalog number: 850457-25 mg)

3. 1,2-Dioleoyl-sn-glycero-3-phosphoethanolamine labeled with Atto 647N (DOPE-Atto647N) (Sigma-Aldrich, catalog number: 42247-1 mg)

4. Atto 488 maleimide (Sigma-Aldrich, catalog number: 28562-1 mg)

5. OptiPrep^TM^ density gradient medium (Sigma-Aldrich, catalog number: D1556)

6. Dithiothreitol (DTT) (Thermo Scientific^TM^, catalog number: R0862)

7. Tris, hydrochloride (Tris-HCl), molecular biology grade (Millipore, catalog number: 648317)

8. Potassium acetate (Sigma-Aldrich, catalog number: 236497)

9. Tris(hydroxypropyl)phosphine (THP) (Millipore, catalog number: 598250)

10. Trichloroacetic acid (Sigma-Aldrich, catalog number: T6399-100G)

11. Acetone (Sigma-Aldrich, catalog number: 179124-500ML)

12. 4× Laemmli sample buffer (Bio-Rad, catalog number: 1610747)

13. TEV protease (2 mg/mL in 50 mM Tris-HCl pH 8.0, 100 mM NaCl, 10% glycerol, 0.2 mM THP, and 300 mM imidazole (prepared in-house but can also be bought from Merck, catalog number T4455-1KU)

14. Chloroform (Honeywell, catalog number: C2432-1L)

15. Laboratory nitrogen gas

16. Water-free dimethyl sulfoxide (DMSO) (Sigma-Aldrich, catalog number: D8418-50ML)

17. 4%–20% Mini-PROTEAN^®^ TGX Stain-Free^TM^ gels, 10 well, 50 μL, with Precision Plus Protein^TM^ unstained standards (Bio-Rad, catalog number: 17000436)

**Solutions**

1. Flotation buffer (see Recipes)

2. Quenching mix (see Recipes)

3. OptiPrep 48 (see Recipes)

4. OptiPrep 30 (see Recipes)

5. OptiPrep 20 (see Recipes)

6. TCA solution (see Recipes)

7. High pH 1× Laemmli buffer (see Recipes)

**Recipes**

**1. Flotation buffer**

50 mM Tris-HCl (pH 7.4), 150 mM potassium acetate, and 0.1 mM THP

**2. Quenching mix**

100 mM DTT in flotation buffer

**3. OptiPrep 48**

16 mL of OptiPrep^TM^ density gradient medium (stock of 60% w/v) and 4 mL of flotation buffer

**4. OptiPrep 30**

10 mL of OptiPrep^TM^ density gradient medium (stock of 60% w/v) and 10 mL of flotation buffer

**5. OptiPrep 20**

6.7 mL of OptiPrep^TM^ density gradient medium (stock of 60% w/v) and 13.3 mL of flotation buffer

**6. TCA solution**

50 g of TCA in 50 mL of distilled water

**7. High pH 1× Laemmli buffer**

250 μL of Tris-HCl (pH 8.5) (500 mM), 500 μL of distilled water, and 250 μL of 4× Laemmli buffer

**Laboratory supplies**

1. Open-top thin-wall ultra-clear tube 4 mL, 11 × 60 mm (Beckman Coulter, catalog number: 344062)

2. BRAND^®^ microcentrifuge tube, 2 mL with lid, transparent (Millipore Sigma, catalog number: Z628034-500EA)

3. Corning^®^ 384-well solid black (Fisher Scientific, catalog number: 09-761-86)

4. Corning^®^ 50 mL centrifuge tubes (Millipore Sigma, catalog number: CLS430828-100EA)

5. Corning^®^ 15 mL centrifuge tubes (Millipore Sigma, catalog number: CLS430791-500EA)

6. Fisherbrand^®^ borosilicate glass tubes 12 × 75 mm (Fisher Scientific, catalog number: 14-961-26)

7. P1000, P200, and P10 pipettes

8. Positive displacement digital microdispenser, 50 μL (Drummond Scientific Company, catalog number: 3-000-550)

9. Positive displacement digital microdispenser, 50 μL (Drummond Scientific Company, catalog number: 3-000-575)

10. Drummond^®^ microdispenser replacement tubes, 50 μL (Drummond Scientific Company, catalog number: 3-000-250-G)

11. Drummond^®^ microdispenser replacement tubes, 100 μL (Drummond Scientific Company, catalog number: 3-000-275-G)

12. Superose^®^ 6 10/300 GL (Cytiva, catalog number: 17-5172-01)

## Equipment

1. SW 60 Ti swinging-bucket rotor (Beckman Coulter, catalog number: 335650)

2. Ultracentrifuge (Beckman Coulter, model: Optima^TM^ L-90K)

3. Fluorescence plate reader (BMG Labtech, model: CLARIOstar Plus)

4. Centrifuge (Eppendorf, model: 5424R)

5. ÄKTA^TM^ pure 25 (Cytiva) or equivalent FPLC system

6. Vacuum oven (Fisher Scientific, model: Vacutherm^®^)

7. Q500 Sonicator^®^ with microtip probes (Qsonica, catalog number: 4418)

## Software and datasets

1. GraphPad Prism v10.0.1

## Procedure

**Option 1. Preparing fluorophore-labeled protein and liposomes**

In general, fluorophore-labeling of proteins can take place on a surface-exposed, non-essential cysteine as described below or, alternatively, on surface-accessible lysines using an NHS-conjugated fluorophore. Note that the maltose-binding protein does not contain any surface-exposed cysteines, which makes the protocol below specific to the 2C part of the fusion protein.

**A. Fluorophore-labeling of poliovirus 2C**

1. Purify the MBP-2C fusion protein until the step before size exclusion chromatography, as described elsewhere [4]. The linker region between MBP and 2C is specifically cleavable using tobacco etch virus (TEV) protease. Pool the protein fractions to be labeled and determine protein concentration from 280 nm absorbance.

2. Dissolve the ATTO 488 maleimide fluorophore in water-free DMSO to get a stock solution of 10 mM. This stock solution can be kept frozen in a well-sealed screwcap tube for multiple uses.

3. Add an equimolar (to the protein) amount of ATTO 488 maleimide from the 10 mM stock solution to 100 μL of flotation buffer. Subsequently, add this solution to the pooled protein fractions, mix by gentle pipetting, and cover the tube with aluminum foil to avoid photobleaching.

4. Incubate at 4 °C overnight.

5. Quench the reaction by adding the quenching mix (the final concentration of DTT should be 25 mM), mixing by gentle pipetting, and incubating at room temperature for 20 min.

6. Pass the labeled protein over a Superose^®^ 6 10/300 GL size exclusion column equilibrated with the flotation buffer. If possible, record chromatograms at 260, 280, and 500 nm to measure both protein and fluorophore absorption. The labeled MBP-2C protein can be expected to elute at an elution volume of 12.5 mL.

7. Calculate the degree of labeling (DOL) of your protein using the following formula:

DOL = (A_500_ * ε_prot_)/((A_280_ – A_500_ * C_280_) * ε_max_)

Absorbance at 500 nm: A_500_

Protein absorbance at 280 nm: A_280_

The molar extinction coefficient of the protein at 280nm: ε_prot_

The molar extinction coefficient of the dye at its maximum wavelength: ε_max_

Correction factor at 280 nm, as stated by the dye manufacturer: C_280_

**B. Preparing liposomes in the form of fluorophore-labeled small unilamellar vesicles (SUVs)**

1. Mix a total of 1 mg/mL lipids containing POPC (75 mol %), POPE (24.9 mol %), and DOPE-Atto 647N (0.1 mol %), all dissolved in chloroform, in a borosilicate glass tube. Chloroform should not be pipetted using standard micropipettors fitted with plastic tips, but instead using microdispensers fitted with replaceable glass capillary tips (as done here) or using Hamilton syringes. Between uses, the glass capillary tip of the microdispenser should be exchanged, and the plunger rinsed in clean chloroform. Note that chloroform is a volatile, toxic, suspected carcinogen and should be handled in a fume hood with appropriate PPE.

**Caution:** When removing the lipid vials from the freezer, do not open them until they reach room temperature, and wipe the vials dry with paper tissue before opening. This will prevent water contamination inside the vial.

2. In the fume hood, carefully direct a stream of nitrogen gas into the borosilicate tube containing the lipids dissolved in chloroform. Make sure to keep the nitrogen gas flow so low that the chloroform evaporates in an ordered way without splashing. An appropriate nitrogen stream can, e.g., be obtained by attaching rubber tubing to a laboratory nitrogen gas outlet and a P1000 pipette tip (with the tip cut to make it wider) to the end of the tubing (fixed to the tubing by Parafilm).

3. Keep the dried lipids overnight in a vacuum oven for complete removal of chloroform.

4. Remove the lipids from the vacuum oven. Add 500 μL of flotation buffer into the mixture to get a 2 mg/mL lipid solution.

5. Cover the tube with parafilm and vortex the mixture to resuspend the lipids in the buffer.

6. Keep the tube in a water bath at 42 °C for 15 min to completely dissolve the lipids and vortex again at the end of the incubation.

7. Transfer the mixture into a microcentrifuge tube.

8. Place the tube on ice and sonicate the mixture with a tip sonicator (10 s pulse, 20 s rest, and 20% amplitude) until a clear solution is observed. Note that the solution will not be completely clear but significantly less turbid than at the start. Note also that the total sonication time required may vary from time to time since the power transfer into the solution is highly sensitive to the exact positioning of the sonicator tip, which is hard to control. For some lipid compositions, a bath sonicator may be a good option.

9. Centrifuge the tube at 21,000× *g* for 20 min at 4 °C in order to pellet metal residues from the sonicator tip as well as any remaining larger lipid aggregates.

10. Remove whatever amount of liposome supernatant that can be pipetted out without disturbing the pellet and discard the rest.

11. Keep the prepared liposomes at 4 °C until use.

**Caution:** The liposomes should be used for a maximum of three days.

*Note: To check the size distribution of the liposomes, dynamic light scattering may be used.*

**C. Vesicle flotation assay; reaction, preparing the tubes, ultracentrifugation**

1. Prepare 100 μL of reaction with a final concentration of 0.8 μM of labeled MBP-2C, 1 mg/mL SUVs, and 8 μM TEV protease.

2. Incubate for 2 h at room temperature.

*Note: The first time this reaction is performed, it is advisable to use SDS-PAGE to check if MBP-2C is completely cleaved in 2 h. If not, increase the time of incubation.*

3. Add 500 μL of OptiPrep 48 to the tube containing the mixture and mix thoroughly.

4. Place this mix at the bottom of an open-top thin-wall ultra-clear tube.

5. Overlay with 800 μL of OptiPrep 30, then a layer of 800 μL of OptiPrep 20, and finally, a layer of 2 mL of flotation buffer.

*Note: Cut the tip of a P1000 tip to broaden the opening. This will make it easier to deposit the lower-percentage OptiPrep solutions on top without disturbing the interface.*

6. Ultracentrifuge the tube with a SW 60 Ti at 364,000× *g* for 3 h at 4 °C.

**D. Vesicle flotation assay: taking out the fraction and preparing the sample for fluorescence measurement**

1. After centrifugation, take out the fractions as described in the graphical overview, starting from the top. Slowly pipette out the solution from the tube, keeping the pipette tip only just submerged under the surface (as shown in the graphical overview).

2. Dilute each fraction to a total volume of 1 mL using flotation buffer.

3. Pipette 3 × 100 μL of each diluted fraction into three wells of a 384-well plate for triplicate readings.

4. Take 100 μL of OptiPrep 48, 30, and 20 as background control.

5. Measure the fluorescence. For proteins: excitation, 510 nm, emission, 530–540 nm. For lipids: excitation, 647 nm, emission, 664–674 nm.

**Option 2. Using unlabeled protein and lipid vesicles**

**Figure 1.**
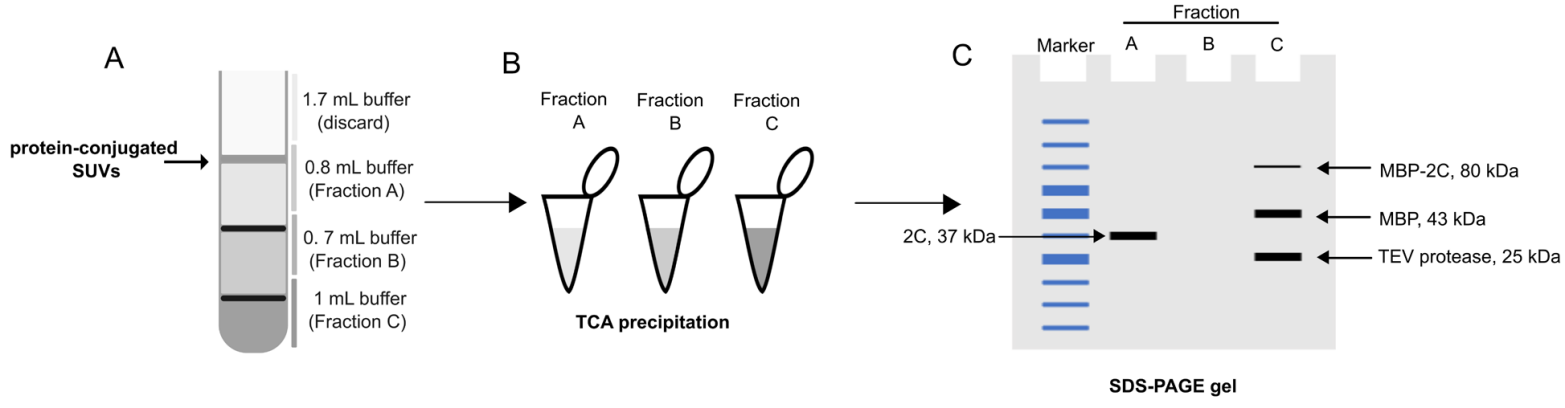
Alternative analysis based on SDS-PAGE. (A) Fractionation of the flotation tube, as for the fluorescence-based readout. (B) Total protein precipitation using trichloroacetic acid (TCA). (C) Analysis of precipitated tubes using SDS-PAGE. A typical pattern of bands is shown.

This procedure does not require the labeling of proteins or liposomes; instead, it uses SDS-PAGE to determine membrane association (Figure 1).

**A. Protein**

Purify the MBP-2C fusion protein as described elsewhere [4]. Perform final size exclusion chromatography run in flotation buffer.

**B. Preparing liposomes in the form of single unilamellar vesicles (SUVs)**

As described above but without the fluorophore.

**C. Vesicle flotation assay: reaction, preparing the tubes, ultracentrifugation**

As described above (Sections C–D of Option 1).

**D. Vesicle flotation assay: taking out the fraction and TCA precipitation of protein**

1. After centrifugation, take out the fractions as shown in the graphical overview.

2. Dilute each fraction to 1 mL using flotation buffer.

3. Add 120 μL of TCA solution to each of the fractions.

**Caution:** As TCA is corrosive, appropriate PPE must be used.

4. Shake the tubes vigorously.

5. Incubate the tubes on ice for 30 min.

6. Centrifuge the tubes at 21,000× *g* for 30 min at 4 °C to pellet the protein.

7. Carefully decant the TCA solution.

8. Add 500 μL of ice-cold acetone to the tube. The acetone wash serves to remove residual TCA that may interfere with downstream gel electrophoresis.

9. Incubate the tubes on ice for 15 min.

10. Centrifuge the tubes at 21,000× *g* for 30 min at 4 °C.

11. Carefully decant the acetone from the tubes.

12. Repeat the acetone wash.

13. Dry the tubes in a fume hood overnight to evaporate any residual acetone.

14. Add 100 μL of high pH Laemmli buffer to each tube and heat the samples at 95 °C for 15 min.

15. Run all samples on an SDS-PAGE gel (e.g., 4%–20% gradient gel) and image the gel with a method of choice (protocol not included). A schematic of the expected outcome is shown in Figure 1C.

## Data analysis

Using labeled proteins and lipid vesicles:

1. Once you have the fluorescence readings, average the technical replicates and subtract the background. Add the background-subtracted, averaged reads from fractions A and B as upper fractions (liposome-bound material). The background-subtracted, averaged fraction C read is the unbound material in solution. From these, the fraction of liposome-bound 2C can be calculated. Other ways of comparing experimental conditions may include, e.g., calculating the protein/lipid ratio in the top fraction, or the fraction of 2C recruited to membranes normalized by the amount of lipid fluorescence in the upper fraction.

2. As quality control, make sure that the lipid fluorescence readings in the upper fraction are consistent across all experiments.

Using unlabeled protein and lipid vesicles: Run an equal amount of each sample on SDS-PAGE. Quantitate gel bands (protocol not provided).

## Validation of protocol

This protocol or parts of it have been used and validated in the research article [4] (Figure 3A–3F, Figure 4A–4C, Figure 5A–5E, and Figure 6A–6I).

In this article, the protocol was used to study and quantify:

- The region of 2C that is important for membrane binding (using truncated constructs of protein and performing flotation assay).
- The different types and sizes of lipid vesicles that 2C prefers (using different combinations of lipids and different sizes of vesicles in the flotation assay).
- The membrane clustering activity of 2C (performing dynamic light scattering on the flotation mixture).
- The activity of 2C on the membrane (performing helicase and nuclease assays on flotation mixture).

## General notes and troubleshooting

**General notes**

1. If adapting the protocol to other proteins, note that the protein purification buffer should not contain an amount of detergent that may compromise the integrity of the liposomes. However, trace amounts of detergent below the critical micellar concentration may be acceptable. Additionally, note that liposomes with specific lipid compositions can be sensitive to the presence of certain divalent metal ions.

2. Verify that the protease cleavage reaction has been completed prior to the flotation assay by running an SDS-PAGE on part of the reaction mix.

3. Ensure complete removal of chloroform when drying the lipid mixture.

4. If using fluorophores that are sensitive to photobleaching, ensure that the tubes are covered with aluminum foil.

5. Stored liposomes should be checked for turbidity; a cloudy solution may indicate aggregation.

6. When layering OptiPrep gradients, pipette gently to avoid mixing the layers of the gradient.

**Troubleshooting**

Problem 1: Low degree of labeling (DOL) of the protein.

Solution: Verify the dye/protein molar ratio and ensure proper mixing during the labeling reaction. If the DOL is still low, there may be water in the DMSO of the fluorophore stock solution, which will lead to hydrolysis of the conjugation group of the fluorophore (maleimide of NHS). Thus, it may be necessary to purchase a new fluorophore and dissolve it in fresh, water-free DMSO.

Problem 2: Liposomes appear cloudy or turbid after sonication.

Solution: Increase the sonication time incrementally while monitoring the sample for clarity. Note that it is normal that the required sonication time varies from one experiment to the next, due to the challenge in controlling the power transfer from the sonicator tip to the solution. Another option is to use a bath sonicator, but for the lipid composition used in this protocol, the bath sonicators we had at hand did not provide sufficient power to clear the solution.

Problem 3: Inconsistent fluorescence readings across replicates.

Solution: Mix fractions thoroughly before pipetting into plates and validate instrument calibration before starting.
